# Deletion of Glutathione Peroxidase-2 Inhibits Azoxymethane-Induced Colon Cancer Development

**DOI:** 10.1371/journal.pone.0072055

**Published:** 2013-08-19

**Authors:** Mike F. Müller, Simone Florian, Stefanie Pommer, Martin Osterhoff, R. Steven Esworthy, Fong-Fong Chu, Regina Brigelius-Flohé, Anna P. Kipp

**Affiliations:** 1 Department Biochemistry of Micronutrients, German Institute of Human Nutrition Potsdam-Rehbruecke, Nuthetal, Germany; 2 Department of Clinical Nutrition, German Institute of Human Nutrition Potsdam-Rehbruecke, Nuthetal, Germany; 3 Department of Radiation Biology, Beckman Research Institute of City of Hope, Duarte, California, United States of America; Yong Loo Lin School of Medicine, National University of Singapore, Singapore

## Abstract

The selenoprotein glutathione peroxidase-2 (GPx2) appears to have a dual role in carcinogenesis. While it protected mice from colon cancer in a model of inflammation-triggered carcinogenesis (azoxymethane and dextran sodium sulfate treatment), it promoted growth of xenografted tumor cells. Therefore, we analyzed the effect of GPx2 in a mouse model mimicking sporadic colorectal cancer (azoxymethane-treatment only). GPx2-knockout (KO) and wild-type (WT) mice were adjusted to an either marginally deficient (−Se), adequate (+Se), or supranutritional (++Se) selenium status and were treated six times with azoxymethane (AOM) to induce tumor development. In the −Se and ++Se groups, the number of tumors was significantly lower in GPx2-KO than in respective WT mice. On the +Se diet, the number of dysplastic crypts was reduced in GPx2-KO mice. This may be explained by more basal and AOM-induced apoptotic cell death in GPx2-KO mice that eliminates damaged or pre-malignant epithelial cells. In WT dysplastic crypts GPx2 was up-regulated in comparison to normal crypts which might be an attempt to suppress apoptosis. In contrast, in the +Se groups tumor numbers were similar in both genotypes but tumor size was larger in GPx2-KO mice. The latter was associated with an inflammatory and tumor-promoting environment as obvious from infiltrated inflammatory cells in the intestinal mucosa of GPx2-KO mice even without any treatment and characterized as low-grade inflammation. In WT mice the number of tumors tended to be lowest in +Se compared to −Se and ++Se feeding indicating that selenium might delay tumorigenesis only in the adequate status. In conclusion, the role of GPx2 and presumably also of selenium depends on the cancer stage and obviously on the involvement of inflammation.

## Introduction

Colorectal cancer (CRC) is the second leading cause of cancer-related death in Western countries. Epidemiological studies linked a sub-optimal selenium status to an increased risk to develop CRC [1]. This is relevant to populations outside of North America like Europe, where average plasma selenium levels are 90 µg/L (reviewed in [2]). Differences in baseline plasma selenium levels of participating subjects may have contributed to the inconsistent results of intervention trials undertaken to study putative anti-carcinogenic properties of selenium supplementation. Accordingly, both the Nutritional Prevention of Cancer (NPC) and the Selenium and Vitamin E Cancer Trial (SELECT) revealed that participants entering the study with plasma selenium levels of ≥120 µg/L did not profit from supplementation [2]. Since selenoprotein P, the most sensitive marker for the selenium status [3], is saturated at 120 µg/L plasma selenium, it is suggested that Se-mediated cancer prevention depends on optimal selenoprotein expression [2].

The selenoproteome consists of proteins encoded by 25 genes in humans [4]. Among them are five selenium-dependent hydroperoxide-reducing glutathione peroxidases (GPx), namely GPx1-4 and GPx6. GPx1-4 are expressed in the murine intestinal epithelium [5], but only GPx2 is predominantly located at the crypt base [6], [7], where also intestinal stem cells reside. In addition, GPx2 is highly expressed in colorectal tumors [6], [8], [9]. The role of GPx2 during CRC development is still unclear. On the one hand, GPx2 decreased tumor numbers in an inflammation-triggered colon carcinogenesis model after application of azoxymethane (AOM) and dextran sodium sulfate (DSS) by acting anti-inflammatory [10]. This is consistent with the spontaneous ileocolitis and intestinal cancer in GPx1/GPx2 double-knockout mice [11], [12]. Both could be nearly completely rescued by one WT allele of GPx2 but not of GPx1 [13]. On the other hand, GPx2 appears to support proliferation. Tumors produced by AOM/DSS treatment were smaller in GPx2-KO than in WT mice [10] as were tumor xenografts derived from GPx2-deficient HT-29 cells compared to control cells [14]. In addition, GPx2 is up-regulated by β-catenin [7], [15] and ΔNp63 [16], both inducing proliferation.

The aims of the current study were to analyze the role of GPx2 and selenium in a model in which CRC is induced solely by AOM, thereby mimicking sporadic colon carcinogenesis in humans [17]. Tumor development was monitored in GPx2-KO and WT mice fed a moderate selenium-deficient (−Se), -adequate (+Se) or -supranutritional (++Se) diet. Since AOM induces activating mutations in β-catenin (reviewed in [18]), co-localization of nuclear β-catenin and enhanced GPx2 expression was analysed.

## Methods

### Animals and Experimental Design

**Figure 1:**
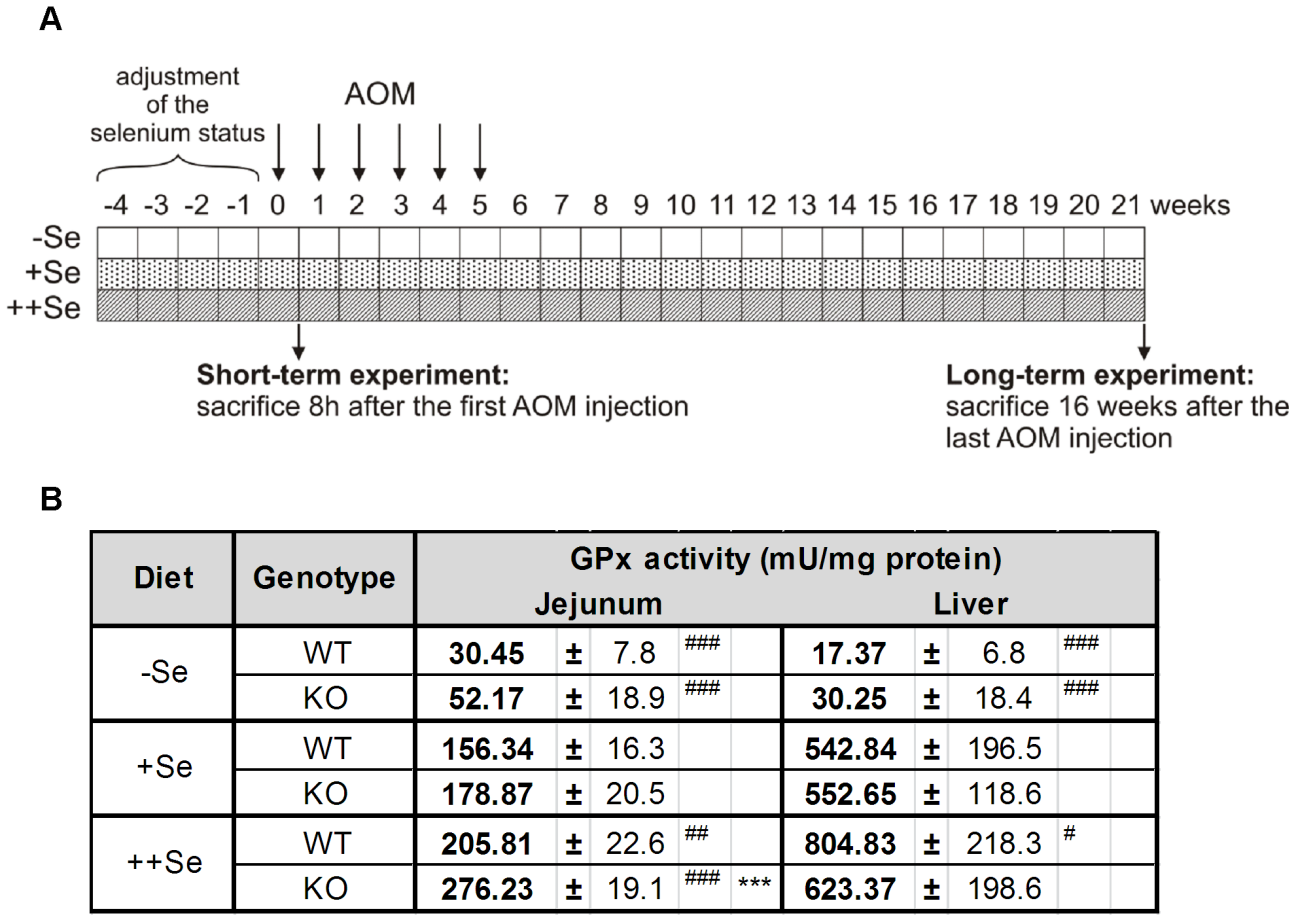
Experimental design and total GPx activity. (A) Weanling mice were fed an experimental diet that was either selenium-poor (−Se), -adequate (+Se) or -supranutritional (++Se) for a minimum of 4 weeks before AOM injection. AOM was injected once a week for six weeks and mice were sacrificed 16 weeks after the last AOM injection (long-term experiment). To study acute AOM effects, mice were killed 8 h after the first AOM injection (short-term experiment). (B) Total GPx activity was determined 8 h after a single saline injection (control groups of the short-term experiment) in the jejunum and liver of GPx2-KO and WT mice. Values are means +SD, n = 5. Significance was calculated using 2-way ANOVA with Bonferroni’s post-test. ***P≤0.001 vs. WT, ^#^P≤0.05, ^##^P≤0.01, and ^###^P≤0.001 vs. +Se.

C57BL/6J WT and GPx2-KO mice, generated as C57BL/6J;129SV/J hybrid had been backcrossed to C57BL/6J mice for five generations [19] before entering the study as littermates. Animals were housed in individually ventilated cages under specific-pathogen-free conditions with a 12 h dark-light cycle and free access to food and water. Mice were fed a torula yeast-based diet (No. C1045, Altromin, Lage, Germany) with a basal selenium content of 0.054 mg per kg diet (−Se). +Se and ++Se diets were produced by adding L(+)-selenomethionine (ACROS Organics, Geel, Belgium) to reach a final selenium concentration of 0.15 mg (+Se) and 0.6 mg (++Se) per kg diet, respectively [20]. The selenium content was measured fluorimetrically as previously described [21]. Weanling mice were fed the experimental diets for 4 weeks to adjust the selenium status before AOM-treatment and then throughout the experiment (Fig. 1A). 10 mg/kg body weight AOM (Sigma, Steinheim, Germany) or an equal volume of saline (Sigma) were injected intraperitoneally once a week for six weeks. Acute effects of AOM were analyzed in 5 mice 8 h after the first AOM injection (short-term experiment). Tumors were analyzed 16 weeks after the last AOM injection (long-term experiment). 20 AOM-treated and 10 saline-treated mice per group were analyzed for tumorigenesis and additional 5 animals were used for histology. The studies were approved by the Governmental Animal Ethics Committee (MLUV 32-2347/4+68). All efforts were made to minimize suffering. Mice were anesthetized with Isofluran® and killed by cervical dislocation. Spleen weights were determined and tissue samples were snap frozen in liquid nitrogen and stored at −80°C.

### Histopathology of Colon Tumors and Preneoplastic Lesions

To identify tumors and preneoplastic lesions, the colon was opened longitudinally, flattened on filter paper and fixed in 4% phosphate-buffered formalin pH 7.0 (Roth, Karlsruhe, Germany). For identification of tumors and aberrant crypt foci (ACF), the colon was stained with 0.1% methylene blue [22]. Afterwards mucin depleted foci (MDF) were identified in the same colon tissue by sequential staining with high-iron diamine (HID) and 1% Alcian blue (AB). The samples were analyzed using a stereo microscope (Olympus SZH10, Olympus Corporation, Tokyo, Japan). The location and diameter of lesions and the number of crypts within an ACF or MDF (crypt multiplicity) were recorded. All MDF consisting of more than two crypts and all tumors were excised and verified by hematoxylin and eosin (H&E) stained sections.

### Immunohistochemistry and Histochemistry

The colon of five AOM-treated animals in the long-term experiment was prepared as Swiss roll for immunohistochemical analyses. To study the short-term effect of AOM, immunohistochemistry (IHC) was performed on the distal colon according to the described protocol [20] with some modifications. Formalin-fixed colon tissues were embedded in paraffin and thin-sectioned (2 µm). After rehydration, tissue sections were microwaved in citrate buffer pH 6.0 (Dako, Glostrup, Denmark) to retrieve antigens. The endogenous peroxidase activity was blocked by incubation in 3% H_2_O_2_ and samples were incubated with primary antibodies overnight at 4°C. The primary antibodies and their dilutions were: anti-β-catenin (#610153, BD Transduction Laboratories™, Lexington, KY, USA; 1∶8.000), anti-ki-67 (M7249, Dako, 1∶60), anti-proliferating cell nuclear antigen (PCNA, #2714-1, Epitomics, Burlingame, CA, USA, 1∶20.000), anti-p53 (NCL-p53-CM5p, Novocastra Laboratories Ltd, Newcastle Upon Tyne, UK, 1∶1.600), anti-GPx2 antiserum [23] (1∶12.000), and anti-F4/80 (MCA497, Serotec, Kidlington (Oxford), UK, 1∶8.000). Secondary antibodies or kits (N-Histofine® Mousestain Kit, N-Histofine® Simple Stain Mouse MAX PO for mouse tissues, anti-rat or anti-rabbit, all from Nichirei Biosciences, Tokyo, Japan), were applied for 30 min at room temperature. Antibody binding was visualized with diaminobenzidine (Dako). Immunoreactivity was scored in a blinded fashion. The length of the PCNA-positive zone and total crypt length was measured using Mirax Viewer Software (Version 1.12.22.0, Carl Zeiss, Göttingen, Germany) from 100 crypts per animal. F4/80-positive cells in the lamina propria along cross-sectioned crypts were scored in 10 crypts per animal.

Quantification of apoptotic cells was performed by means of morphological criteria in hematoxylin stained sections of the distal colon in 200 longitudinally opened crypts per animal as described [20]. To identify MDF in tissue sections, mucins were visualized by sequential staining with 1% AB, pH 2.5 and Periodic Acid-Schiff (PAS) and counterstaining with hematoxylin. Serial sections were stained with PAS/AB, H&E, β-catenin, ki-67, and GPx2 individually. β-Catenin and GPx2 immunoreactivity was scored with respect to subcellular localization and position in the crypt. An enhanced immunoreactivity was defined as either an enhanced maximal staining intensity compared to adjacent normal tissue or an enlargement of the positive area in cells or crypts or both.

### Total GPx Activity

The GPx activity was measured in a glutathione reductase-coupled test [24] that was modified for 96-well microtiter plates as described [20] using a plate absorbance reader (Synergy2 Microplate Reader, BioTek, Bad Friedrichshall, Germany) at 340 nm. Total GPx activity was calculated from µmol NADPH consumption per minute according to Lambert-Beer’s law and expressed as mU/mg protein.

### Statistical Analysis

Comparing two groups, significant differences were calculated by unpaired Student’s t-test. For comparing more than two groups, 2-way ANOVA followed by Bonferroni’s post-test was used (GraphPad Prism® version 5.0, San Diego, CA, USA). Tumor incidence was analyzed with contingency tables using Fisher’s exact test (SPSS, version 16, IBM). To consider tumor numbers, contingency tables were calculated for tumor incidence weighted with tumor number. A p-value of <0.05 was regarded as statistically significant.

## Results

### GPx2-KO Mice Develop Fewer AOM-induced Tumors than WT Mice Under Both, a Selenium-poor and -supplemented Diet

After feeding mice the −Se and ++Se diets, jejunal and hepatic GPx activity was significantly decreased and increased, respectively, compared to +Se groups (Fig. 1B). Higher GPx activity was found in the jejunum of GPx2-KO compared to WT mice on ++Se diet (Fig. 1B), presumably caused by a compensatory up-regulation of GPx1 as we previously described for ileum and colon [20]. The intestinal localization of GPx2 was analyzed by IHC in the colon of WT mice (Fig. S1A). GPx2 was highest at crypt bases and undetectable at the upper third of crypts. Similar levels of GPx2 protein were detected in the colon of WT mice on +Se and ++Se diets, while GPx2 expression was down-regulated on −Se diets.

**Figure 2:**
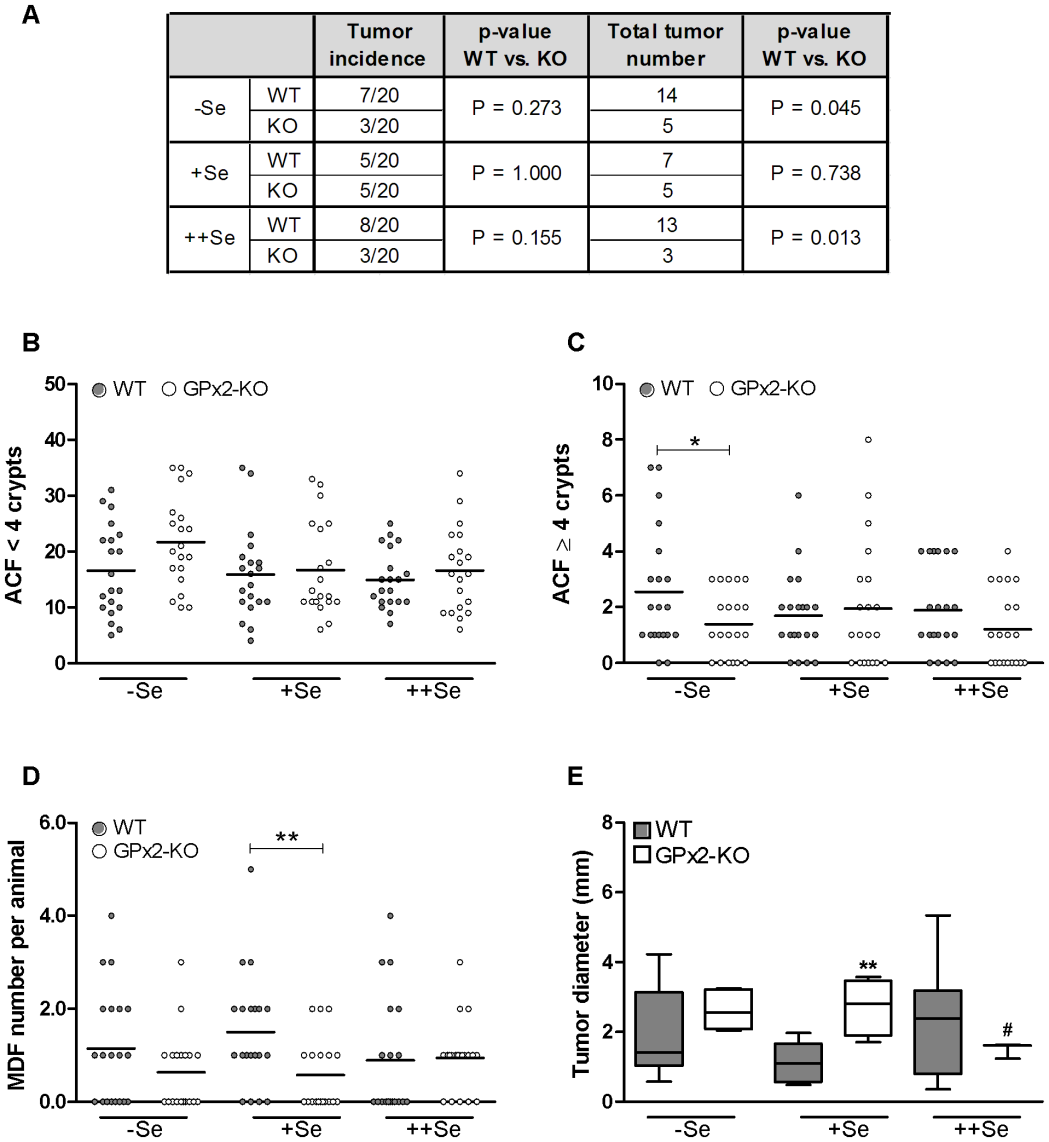
AOM-induced adenomas, ACF, and MDF in WT and GPx2-KO mice at different selenium states. 16 weeks after the last AOM injection WT and GPx2-KO mice were analyzed for tumor incidence and total tumor number per group (A) and for ACF consisting of <4 crypts (B) or ≥4 crypts (C) by methylene blue staining. MDF (D) were counted in the HID/AB stained colon. ACF and MDF numbers are shown as scatter dot plots with mean. Tumor diameter is depicted as box and whiskers from min to max (E). P-values for (A) were calculated using Fisher’s exact test. To test significance for total tumor number, tumor incidence was weighted with tumor number. P-values for (B–E) were calculated using unpaired Student’s t-test. *P≤0.05 and **P≤0.01 vs. WT, ^#^P≤0.05 vs. −Se. No ACF, MDF or tumors were observed in saline-treated controls (n = 10).

In order to test the effect of a GPx2-KO on tumor development, mice were challenged six times with AOM and analyzed 16 weeks after the last AOM application. Although tumor incidence was not significantly different between the experimental groups, −Se and ++Se GPx2-KO mice had significantly lower tumor numbers than WT mice on the same diets (Fig. 2A). While tumor numbers were similarly low in GPx2-KO mice irrespective of the selenium status (−Se: 5, +Se: 5, ++Se: 3), tumor numbers in WT mice were higher in the −Se and ++Se groups than in the +Se group (−Se: 14 and ++Se: 13 vs. +Se: 7). The selenium status did not significantly affect tumor incidence or total tumor number. All tumors were non-invasive adenomas located from the transverse colon to the rectum [25].

We quantified ACF and MDF, which both are considered as preneoplastic markers [26]. We differentiated between ACF with a low (<4) and a high (≥4) crypt multiplicity because the latter is supposed to be a better biomarker for tumor development than the former [26]. Numbers of ACF were not affected by the selenium diets (Fig. 2B and C). While −Se WT mice tended (p = 0.06) to have fewer ACF with low crypt multiplicity than respective GPx2-KO mice (Fig. 2B), −Se WT mice had significantly more ACF with high crypt multiplicity than GPx2-KO mice (Fig. 2C). We also quantified MDF, which are characterized by changes in the cellular composition of the crypt including loss of goblet cells. In contrast to ACF, MDF always consist of dysplastic crypts likely developing into tumors [26], [27]. Only +Se WT mice had significantly more MDF compared to GPx2-KO mice (Fig. 2D) while −Se and ++Se WT mice had more tumors than GPx2-KO mice (Fig. 2A). Thus, in selenium-adequacy the difference between genotypes was only detected at the MDF level. Tumor numbers were equally low in both genotypes under +Se which might implicate that the conditions for tumor development were more appropriate in −Se and ++Se WT groups.

Tumor diameter was measured as a parameter for tumor growth. GPx2-KO and WT mice on −Se and ++Se diets had similar tumor size, whereas tumors were significantly larger in +Se GPx2-KO than in +Se WT mice (Fig. 2E). ++Se GPx2-KO mice had smaller tumors compared to −Se GPx2-KO mice. In WT groups, dietary selenium did not significantly affect tumor size.

### GPx2 and β-catenin were Elevated in Preneoplastic Lesions

GPx2 expression is increased especially during early stages of neoplastic transformation in the human intestine [6] which might be mediated by β-catenin [7]. To test whether GPx2 expression is also increased in preneoplastic lesions of AOM-treated mice, GPx2 was analyzed by IHC in +Se WT mice 16 weeks after the last AOM application. The PAS/AB staining identified 67 MDF in five mice. Serial sections were stained for β-catenin, GPx2, and ki-67. β-Catenin immunoreactivity was increased in 85% (57/67) of the MDF in comparison to the surrounding normal tissue in agreement with published data [27]. GPx2 immunoreactivity was increased in 67% (45/67) of the MDF compared to normal crypts.

**Figure 3:**
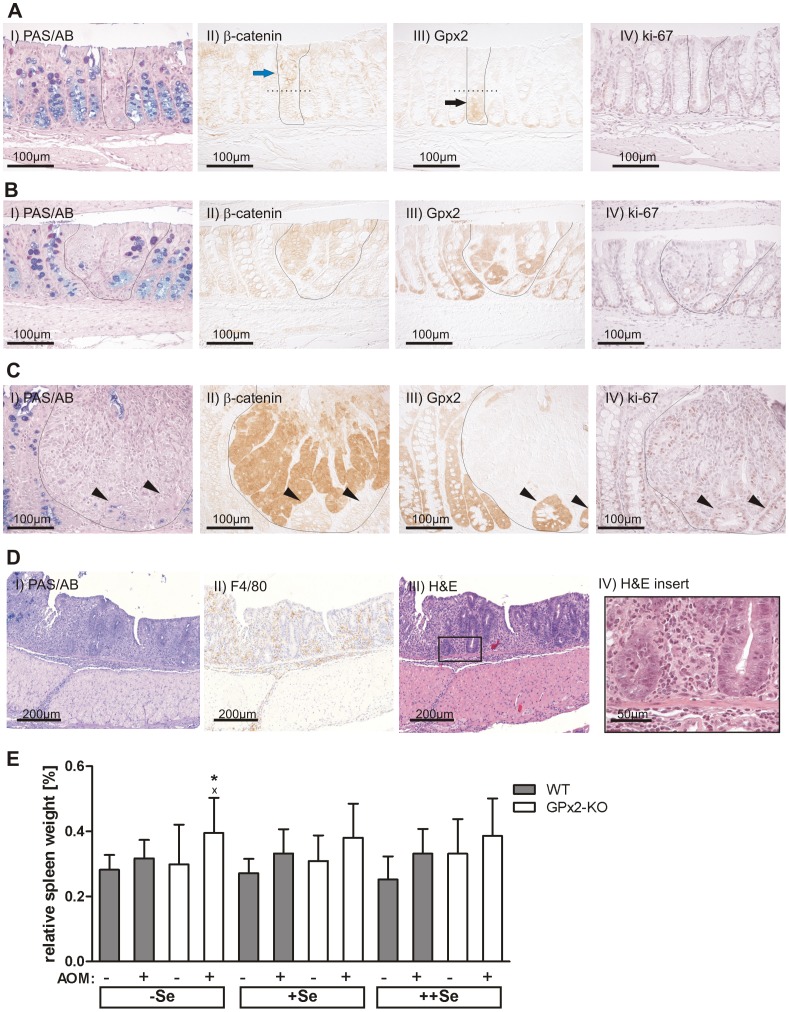
Colon IHC staining and spleen weights of mice 16 weeks after the last AOM-treatment. Serial IHC staining of colon Swiss rolls was performed for PAS/AB, β-catenin, GPx2, and ki-67 in five +Se WT mice 16 weeks after the last AOM-treatment (A–C). Representative stainings of an MDF characterized by a minor grade of dysplasia and low crypt multiplicity (A), an MDF with advanced dysplasia and higher multiplicity (B), and one microadenoma (C). Florid inflammation in AOM-treated, −Se GPx2-KO mice was identified by PAS/AB, F4/80, and H&E staining (D). Spleen weights were normalized to body weight (E). Means +SD, n = 10 in saline-treated and n = 20 in AOM-treated groups. Significance was determined using 2-way ANOVA with Bonferroni’s post-test. *P≤0.05 vs. WT, ^x^P≤0.05 vs. saline.

MDF were further characterized based on their grade of dysplasia and crypt multiplicity to determine whether GPx2 and β-catenin co-localize. MDF with a minor grade of dysplasia had a nearly normal crypt morphology, a low multiplicity, some remaining goblet cells (Fig. 3A-I), and a normal distribution of ki-67-positive cells (Fig. 3A-IV). These MDF exhibited more membrane-localized β-catenin, which was predominantly detected in the luminal part (Fig. 3A-II blue arrow) of the crypt or in entire crypts compared to normal crypts. GPx2 immunoreactivity was slightly increased at the crypt base of those MDF compared to normal crypts and extended towards the luminal side (Fig. 3A-III black arrow). Therefore, in MDF with minor dysplasia GPx2 and β-catenin are elevated in different epithelial cells.

MDF with advanced dysplasia were characterized by higher multiplicity, thickened epithelial cells, nearly complete loss of goblet cells, and enhanced ki-67 staining (Fig. 3B-I and IV) and exhibited both more β-catenin and GPx2 immunoreactivity in the entire crypt (Fig. 3B-II and III). At the cellular level, GPx2 and β-catenin co-localized in the middle of MDF with advanced dysplasia, suggesting that high levels of both proteins were only detectable in a specific area of the crypt during a specific time of dysplastic transformation.

Furthermore, one microadenoma was characterized by complete goblet cell loss, disturbed crypt architecture (Fig. 3C-I), massive up-regulation of nuclear and cytoplasmic β-catenin (Fig. 3C-II), and a high number of ki-67-positive cells (Fig. 3C-IV). In this structure, GPx2 expression was completely lost in the area with elevated β-catenin, while it remained up-regulated in less dedifferentiated surrounding crypts (Fig. 3C-II and III, arrow heads) with similar characteristics as MDF with advanced dysplasia.

### AOM-treated Selenium-poor GPx2-KO Mice Developed a Florid Inflammation in the Colon

AOM-treated −Se GPx2-KO mice developed a clear inflammation ranging from a moderate to a florid state, which was not observed in any other group (Fig. 3D). In the distal colon, inflammation was moderate as characterized by crypt degeneration, infiltration of immune cells (shown by F4/80-positive myeloid cells), and an increase in connective tissue. At the beginning of the transverse colon a florid inflammation (Fig. 3D) with massive immune cell infiltration was observed. Crypt architecture was distorted in the affected areas probably due to failure of epithelial cell renewal.

A well-established indicator for systemic inflammation is spleen weight. Spleen weight tended to be increased in all AOM-treated groups (Fig. 3E) and was significantly higher in AOM-treated −Se GPx2-KO mice than in respective WT mice. This correlates with colonic inflammation. Due to the massive distortion in epithelial morphology, β-catenin and GPx2 co-localization in −Se GPx2-KO mice could not be compared to the other groups. No overt differences in dysplastic structures or corresponding β-catenin levels were observed between +Se and ++Se GPx2-KO and WT mice (data not shown).

### AOM-treatment Increased Apoptotic Cell Death in Epithelial Cells of GPx2-KO Mice

**Figure 4:**
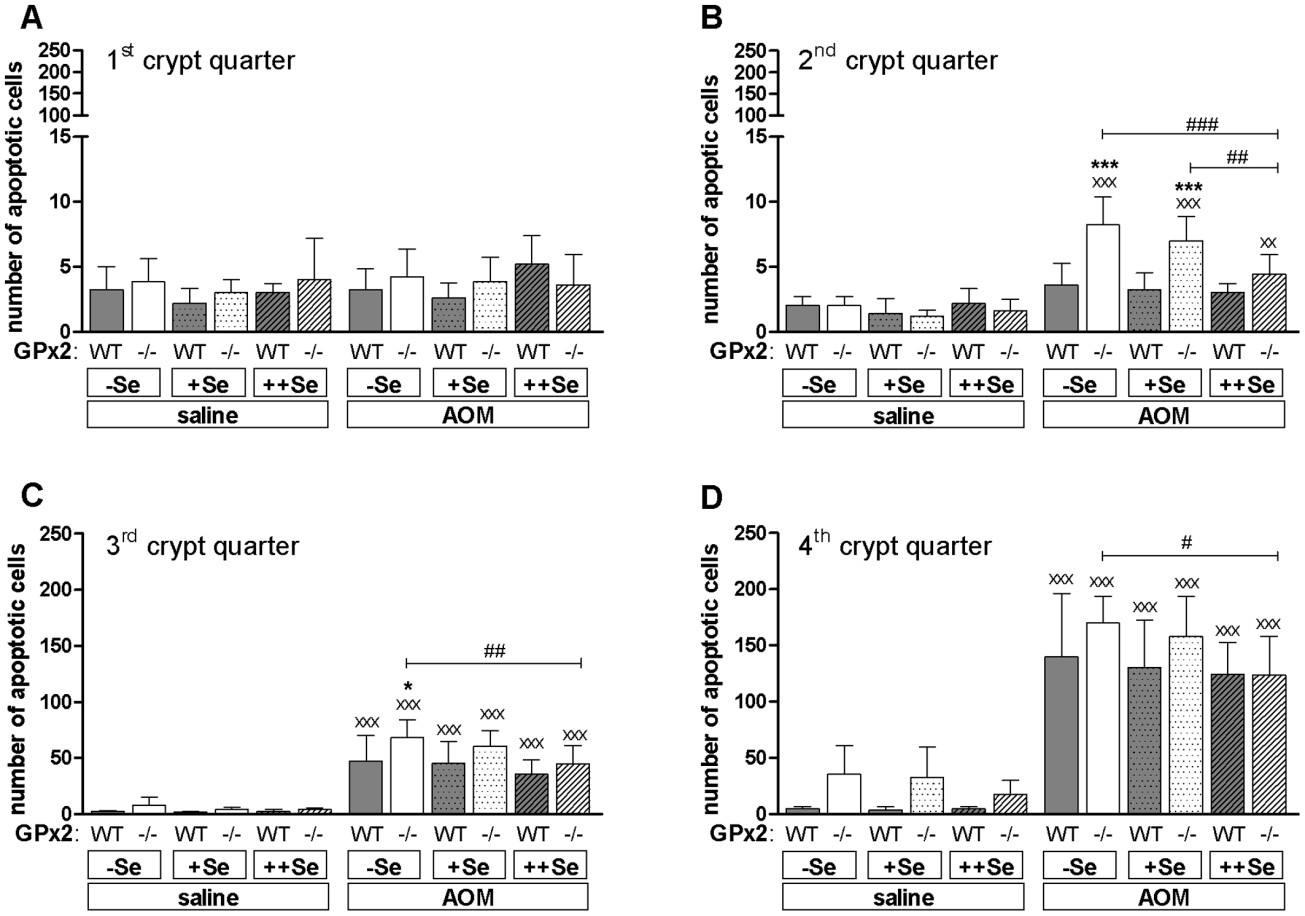
Numbers of AOM-induced apoptotic epithelial cells in GPx2-KO and WT mice. (A–D) 8 h after AOM or saline injection, apoptotic epithelial cells were counted in 200 crypts per animal using H-stained sections of the distal colon. Crypts were divided into 4 quarters. The first indicates the upper part of the crypt and the fourth the crypt base. Data are shown as means +SD, n = 5. Significance was determined using 2-way ANOVA with Bonferroni’s post-test. *P≤0.05, ***P≤0.001 vs. WT, ^#^P≤0.05, ^##^P≤0.01, ^###^P≤0.001 as indicated, ^xx^P≤0.05, ^xxx^P≤0.001 vs. saline.

AOM is known to induce DNA alkylation adducts that peak 8 h after its application [28]. The capacity to remove DNA adducts by DNA repair or p53-dependent apoptosis of the damaged cells is critical to the prevention of cancer development [29]. As previously reported, the number of apoptotic cells at the crypt base is significantly higher in GPx2-KO than in WT mice and is dose-dependently diminished by increasing the selenium supply [20]. It is well established that short-term AOM-treatment increases apoptosis [28]. We counted apoptotic cell numbers (Fig. 4A-D) and analyzed nuclear localization of p53 (Fig. S1B and C) 8 h after AOM injection. As expected, nuclear staining of p53 at the crypt base was increased in all AOM-treated mice (Fig. S1B and C), whereas it was undetectable in mice without AOM-treatment (Fig. S1C). AOM did not modify GPx activity (data not shown) or GPx2 expression in WT mice (Fig. S1A).

AOM-treatment did not affect apoptotic cell numbers in the luminal 1^st^ crypt quarter (Fig. 4A) irrespective of the genotype or selenium status. GPx2-KO mice had significantly more AOM-induced apoptotic cells in −Se and +Se groups of the 2^nd^ crypt quarter (Fig. 4B) and in the −Se group of the 3^rd^ crypt quarter (Fig. 4C) than respective WT mice. In the 4^th^ crypt quarter, the number of AOM-induced apoptotic cells was equally high in both genotypes (Fig. 4D). The increased number of apoptotic cells at the crypt base of untreated GPx2-KO mice could be confirmed (Fig. 4D, saline). In summary, GPx2-KO mice had more AOM-induced apoptotic cells than WT mice that are specifically located in the middle of the crypt, the 2^nd^ and 3^rd^ crypt quarter. Thus, AOM-initiated cells might have been eliminated more efficiently in GPx2-KO mice resulting in the development of fewer tumors.

### Crypt Length and Proliferative Zone are Enlarged in the Colon of GPx2-KO Mice

**Figure 5:**
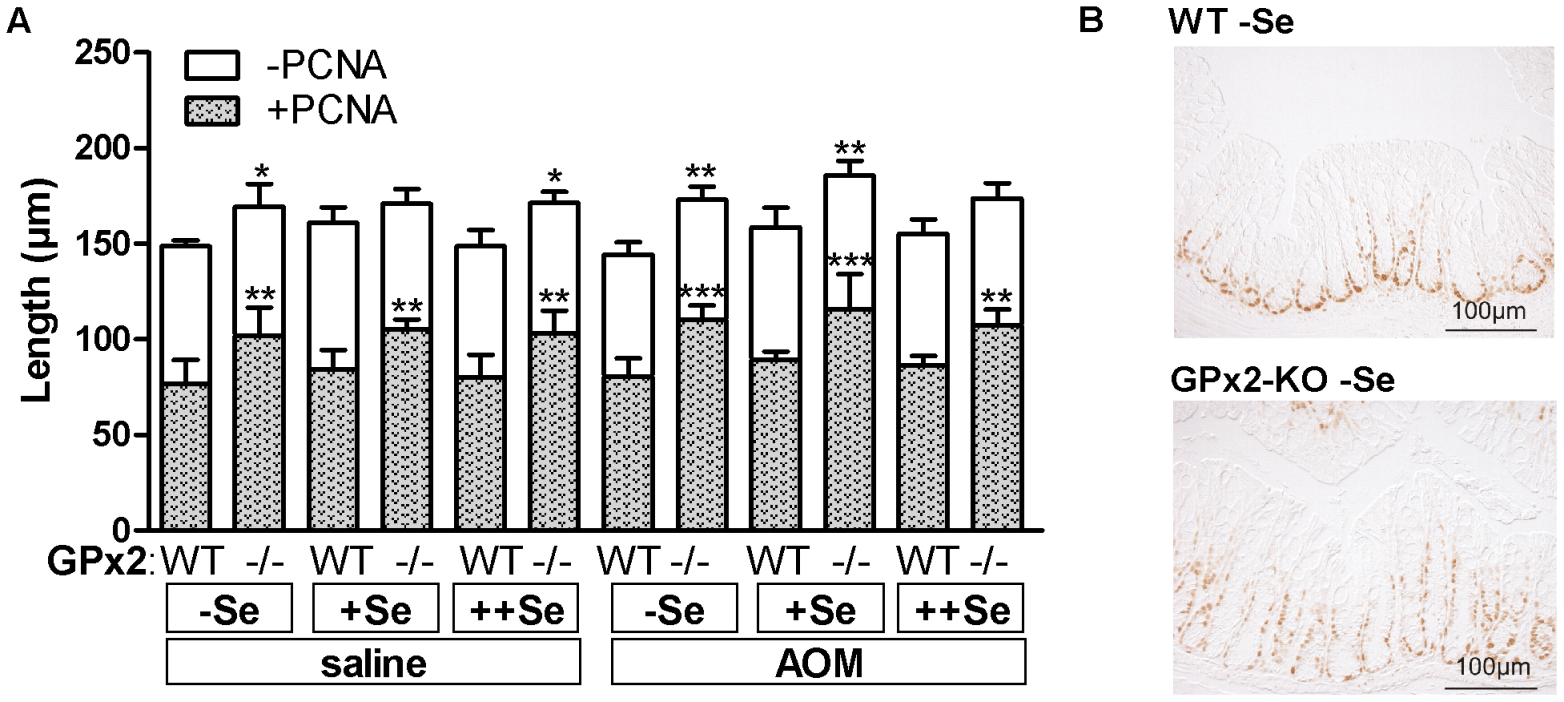
Increased length of the crypt and PCNA-positive zone in the colon of GPx2-KO mice. The length of crypts and the PCNA-positive zone in the distal colon of GPx2-KO and WT mice fed the different selenium diets was determined 8 h after a single dose of AOM or saline. (A) The length of the PCNA-positive zone (grey colour) and PCNA free zone (white colour) were measured in the distal colon. Entire bars represent total crypt length. Values are means +SD, n = 5. Significance was calculated using 2-way ANOVA with Bonferroni’s post-test. *P≤0.05, **P≤0.01, ***P≤0.001 vs. WT. Symbols above the grey bars indicate differences in the PCNA-positive zone. Symbols above the white bars indicate differences in total crypt length. (B) Representative PCNA staining of saline-treated, −Se WT and GPx2-KO mice.

To analyze whether the changes in apoptotic cells could further affect the morphology of the crypt, its length and the height of the proliferative PCNA-positive zone was measured. Both were significantly increased in GPx2-KO mice irrespective of the selenium status or AOM-treatment (Fig. 5). The enlargement of the PCNA-positive zone persisted after normalization for crypt length (data not shown), meaning that in GPx2-KO mice the proliferative zone covered ∼10% more of the total crypt length than in the WT. Since AOM only induces apoptosis in proliferating cells [28], the AOM-induced apoptotic cells should be co-localized with proliferative PCNA-positive cells. The IHC for PCNA of GPx2-KO mice revealed that the proliferating cells did not only reside in the 4^th^ and 3^rd^ crypt quarter as observed in WT mice, but also in the 2^nd^ crypt quarter (Fig. 5B). This correlated with more apoptotic cells in the 2^nd^ crypt quarter in −Se and +Se GPx2-KO mice (Fig. 4B).

### GPx2-KO Mice are Characterized by a Low-grade Intestinal Inflammation

**Figure 6:**
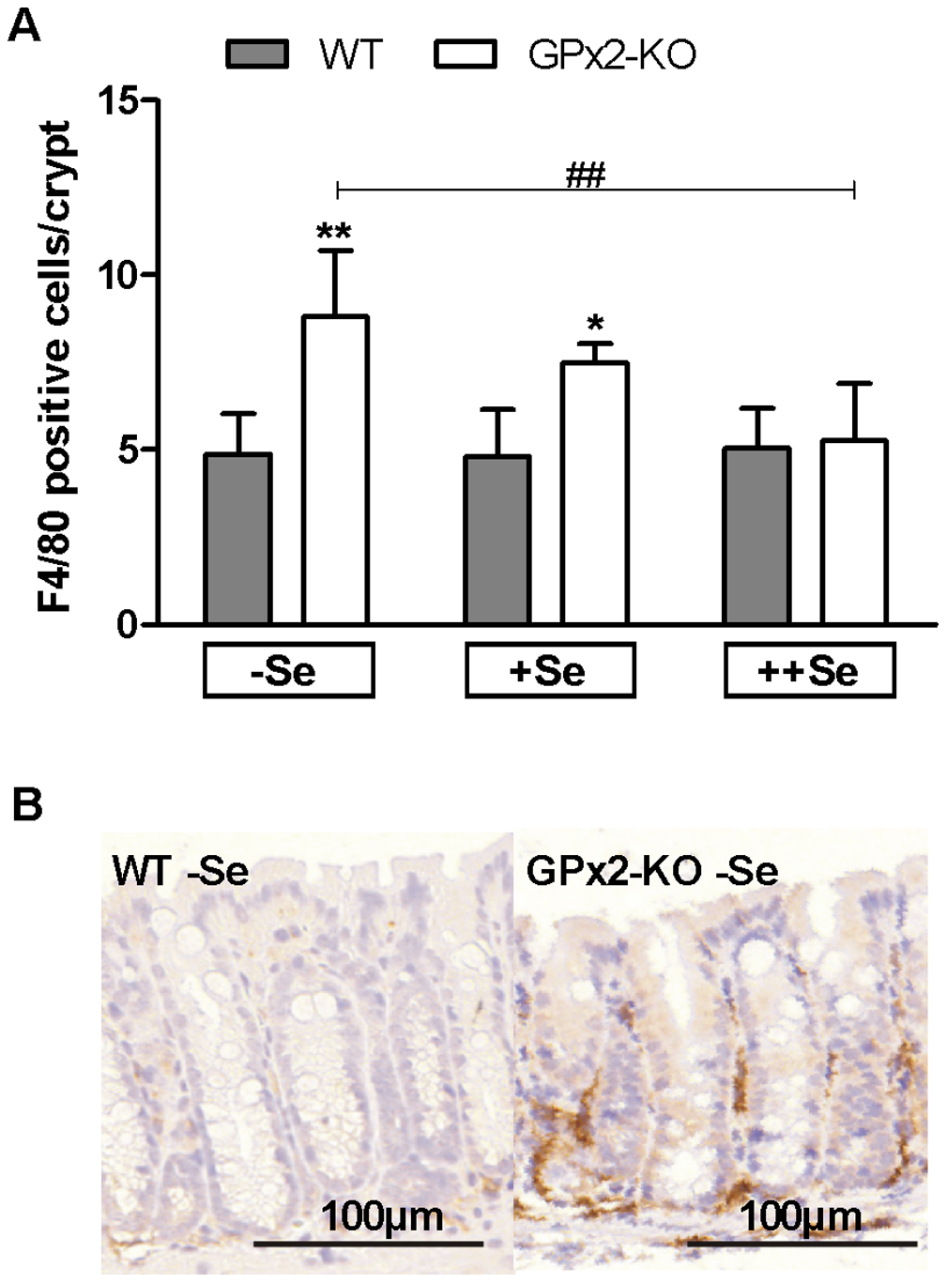
More intra-epithelial myeloid cells in untreated GPx2-KO mice. F4/80-positive myeloid cells were analysed in untreated GPx2-KO and WT mice after 7 weeks on the selenium diets. F4/80-positive cells in the lamina propria adjacent to a crypt were counted in the colon (A). Means+SD, n = 4. Representative F4/80 staining of the −Se WT and GPx2-KO colon (B). Significance was determined using 2-way ANOVA with Bonferroni’s post-test. *P≤0.05, **P≤0.01 vs. WT, ^##^P≤0.01 vs. −Se.

Previous analyses have shown that a double knockout of GPx1 and GPx2 results in spontaneous ileocolitis [11]. To characterize the basal intestinal inflammation status in untreated GPx2-KO mice, F4/80-positive myeloid cells invading the lamina propria of the colonic epithelium were counted. Genotype differences were again highest in −Se groups, decreased in +Se and disappeared in ++Se groups (Fig. 6A). In addition, −Se GPx2-KO mice had more CD3-positive T-cells in the lamina propria than WT mice (data not shown). Whereas AOM-treated −Se GPx2-KO mice developed a florid inflammation with disturbed crypt architecture (Fig. 3D), untreated −Se GPx2-KO mice exhibited less severe inflammation and maintained an intact crypt morphology (Fig. 6B). In summary, GPx2-KO mice developed a basal low-grade inflammation, which was highest in −Se.

## Discussion

### Tumor Development and Initiation

GPx2-KO mice developed fewer tumors on −Se or ++Se diets and fewer MDF on the +Se diet than respective WT mice (Fig. 2A and D). This indicates that GPx2-KO mice are more resistant to AOM-induced cancer. In contrast, GPx2-KO mice developed more tumors in an AOM/DSS-induced colon cancer model [10], which correlated with a more severe DSS-colitis upon GPx2 deletion. Thus, the anti-carcinogenic activity of GPx2 in inflammation-triggered colon cancer is mainly caused by its anti-inflammatory role. The pro-carcinogenic activity of GPx2 after AOM-treatment alone highlights the crucial impact of the DSS-colitis as a tumor-driving force in the AOM/DSS model. AOM induces G/A base conversions which mainly result in activating mutations in β-catenin. These mutations are counteracted by defense mechanisms including DNA base repair or apoptosis. Therefore, apoptosis plays a predominant role in the prevention of AOM-induced cancer. At 6–8 h post-AOM treatment, crypt epithelial cells undergo apoptosis in a p53-dependent manner [30], [31] (Fig. S1B and C). If damaged progenitor cells evade apoptosis, they may produce aberrant crypts, which can develop into tumors [32]. Mice deficient in pro-apoptotic genes like *p53* [33], *Bak* [34], and *Puma* [35] have higher numbers of ACF or tumors and a lower rate of AOM-induced apoptosis.

We have shown here and previously that GPx2 deficiency increases the basal apoptosis rate in crypt bases of the intestinal epithelium regardless of selenium status, although most evidently in the −Se state (Fig. 4D, saline) [20]. Another characteristic of the GPx2-KO intestinal epithelium is an enlarged proliferation zone (Fig. 5) which presumably is an attempt to counteract apoptotic cell loss. 8 h after AOM-treatment, more AOM-induced apoptotic cells were counted in the mid-crypt region (2^nd^ and 3^rd^ crypt quarter; Fig. 4B and C) of −Se and +Se GPx2-KO in comparison to WT mice. Only GPx2-KO mice have a higher number of proliferating cells in this region. Higher levels of basal and AOM-induced apoptosis in GPx2-KO mice are supposed to efficiently eliminate AOM-initiated cells. In WT mice, dysplastic crypts are characterized by increased GPx2 levels (Fig. 3A and B) which presumably suppress apoptosis. In GPx2-KO dysplastic crypts an enhanced rate of apoptosis might counteract malignant progression. Based on these results, GPx2 obviously is able to enhance cancer development by suppressing apoptosis which otherwise eliminates damaged or transformed cells.

### Tumor Size and Promotion

Unexpectedly, tumors were larger in +Se GPx2-KO than in WT mice (Fig. 2E). This does not fit with the pro-proliferative function of GPx2 as postulated from a xenograft model with siRNA-mediated knockdown of GPx2 [14] and AOM/DSS-treated GPx2-KO mice [10], which would have resulted in larger tumors in the WT rather than in the GPx2-KO. In the present study, tumor size was equal in −Se and +Se GPx2-KO mice and significantly decreased with ++Se (Fig. 2E). This correlated with the amount of infiltrated inflammatory cells (Fig. 6) in the untreated GPx2-KO colon, which was only increased in −Se and +Se but not in ++Se GPx2-KO in comparison to WT mice. Mice deficient in both GPx1 and GPx2 spontaneously develop ileocolitis [11] and DSS-induced colitis is more severe in mice deficient in GPx2 [10]. Here, we demonstrate that unchallenged GPx2-KO mice have a low-grade colitis, characterized by increased spleen weight (Fig. 3E), infiltration of inflammatory cells (Fig. 6), and crypt elongation (Fig. 5A). In addition, plasma TNFα levels were increased in +Se GPx2-KO mice (unpublished observation). Genes of pro-inflammatory cytokines are up-regulated during tumorigenesis [36] to produce a tumor-promoting environment. During DSS-induced colitis NF-κB-regulated cytokines like IL-6 or TNFα are massively activated [36], [37]. Therefore, changes in single mediators are negligible in the AOM/DSS model. For instance the deletion of COX-2 did not affect tumor numbers in the AOM/DSS model, but completely abolished tumor development in only AOM-treated mice [38]. This indicates that small changes in the inflammatory environment are critical in the AOM model where pro-inflammatory stimuli are needed to support tumor promotion [39]. Basal low-grade inflammation might thereby explain why tumors were larger in AOM-treated but smaller in AOM/DSS-treated GPx2-KO mice compared to respective WT mice.

In −Se GPx2-KO mice, inflammation was further aggravated by AOM-treatment. This resulted in higher spleen weights in relation to mice without AOM-treatment (Fig. 3E) and often in florid inflammation (Fig. 3D). Even though inflammation was most severe under −Se, −Se GPx2-KO mice had the same tumor size as −Se WT mice. This can be explained by selenium effects on tumor size in the WT. Tumors tended to be larger in −Se and ++Se compared to +Se groups (Fig. 2E, p<0.07). Based on xenograft experiments growth-promoting properties have been anticipated for GPx2. In the present study, proliferation-supportive functions of GPx2 are not directly obvious but cannot be ruled-out because they might have been overridden by the pro-inflammatory microenvironment found in GPx2-KO mice.

Independent of multiplicity or grade of dysplasia, GPx2 immunoreactivity was increased in 67% of the analyzed MDF (Fig. 3A and B), indicating that GPx2 expression might be part of a proliferation-supportive signature activated in early lesions. However, at the cellular level, GPx2 and β-catenin appear to only co-localize in specific areas of advanced MDF and at specific points in time during their transformation. Thus, additional transcription factors besides β-catenin [7] might be involved in GPx2 up-regulation at earlier stages. We found one microadenoma, which was characterized by a massive up-regulation and nuclear localization of β-catenin and complete loss of GPx2 (Fig. 3C). This down-regulation of GPx2 in areas of advanced dedifferentiation fits with the postulated only transient up-regulation of GPx2 during cancer development [6], [40].

In this study, the promotion from MDF to adenomas appeared to be slower in +Se than in −Se or ++Se WT mice making differences between genotypes in the +Se status only detectable at the level of MDF (Fig. 2D). Therefore, we propose that selenium-adequacy, but not deficiency or supplementation, retards tumorigenesis in WT mice.

### Selenium, Selenoproteins and Cancer Development

This study aimed to analyze changes in the selenium supply which are achievable by normal nutrition. Similar studies analyzing selenium diets in solely chemically-induced murine cancer models are limited. In most cases much higher selenium concentrations or other selenium sources were supplied. To study the role of different selenoproteins during cancer development, mice carrying a mutation in the gene for the selenocysteine transfer RNA (i^6^A mice) and, thus, unable to synthesize stress-related selenoproteins like GPx1 were exposed to the AOM model [41]. These mice revealed a substantially higher number of ACF than WT mice indicating that stress-related selenoproteins play a major role in cancer prevention by selenium. However, overexpression of GPx1 enhanced skin tumor growth [42]. Here, intestinal GPx activity was concentration-dependently increased with increasing supply of selenium (Fig. 1B). As both, GPx1 down-regulation [41] and GPx1 overexpression [42] support tumor development, GPx1 could be responsible for the similar −Se and ++Se effect on tumor development in the WT. In addition, it is important to keep in mind that GPx1 is up-regulated most probably to compensate for loss of GPx2 (Fig. 1B), which might also influence carcinogenesis in GPx2-KO mice. Another candidate to explain selenium-dependent effects is TrxR1 which, depending on the context, either acts pro- or anti-carcinogenic [43], [44]. Similar to GPx, TrxR activity was further increased in ++Se WT mice [10], indicating that high levels of TrxR1 might support tumor growth. SepW1 mRNA expression was identified as highly sensitive biomarker for the selenium status [5] and indeed was concentration-dependently increased from −Se to +Se to ++Se (data not shown). The knowledge about the *in vivo* role of SepW1 during cancer development is limited. Cell culture studies revealed that SepW1 is required for cell cycle progression, especially regulating G1- to S-phase transition [45]. Sep15 [46] and GPx3 [47] are additional selenoproteins which have substantial roles during cancer development. Therefore, further studies are essential to completely understand the role of all selenoproteins to explain selenium effects on cancer development.

### Conclusion

In summary, GPx2-KO mice developed fewer AOM-induced tumors and MDF than WT mice. The decrease in tumor development could be attributed to the efficient elimination of damaged or premalignant epithelial cells in GPx2-KO mice. GPx2 was up-regulated in dysplastic WT crypts, where it appears to act anti-apoptotic and, thus, supports tumor development. Larger tumors in the GPx2-KO were linked to a tumor-promoting environment characterized as low-grade inflammation. Selenium-adequacy is supposed to retard tumor development in comparison to a marginal deficiency but also to a supra-nutritional selenium supply. Taken together, the putative effect of GPx2, other selenoproteins, and probably selenium itself, essentially depends on the cancer stage and the involvement of inflammation.

## Supporting Information

Figure S1 **GPx2 and p53 response to AOM 8 h after single treatment.** (A) GPx2 IHC staining was scored in the colon of WT mice fed the different selenium diets (nd = not detectable). (B) Nuclear staining of p53 was scored in WT and GPx2-KO mice. (C) Representative picture of p53 in -Se WT and GPx2-KO colon. Values are means +SD, n = 5. Significance was calculated using 2-way ANOVA with Bonferroni’s post-test. ^#^P≤0.05, ^##^P≤0.01, ^###^P≤0.001 vs. -Se, ^xx^P≤0.01, ^xxx^P≤0.001 vs. saline.
(TIF)
