# Supplementary figures and images for: Deletion of Glutathione Peroxidase-2 Inhibits Azoxymethane-Induced Colon Cancer Development
Source: PLoS One. 2013 Aug 19;8(8):e72055. doi: 10.1371/journal.pone.0072055 (PMC3747154; doi:10.1371/journal.pone.0072055)

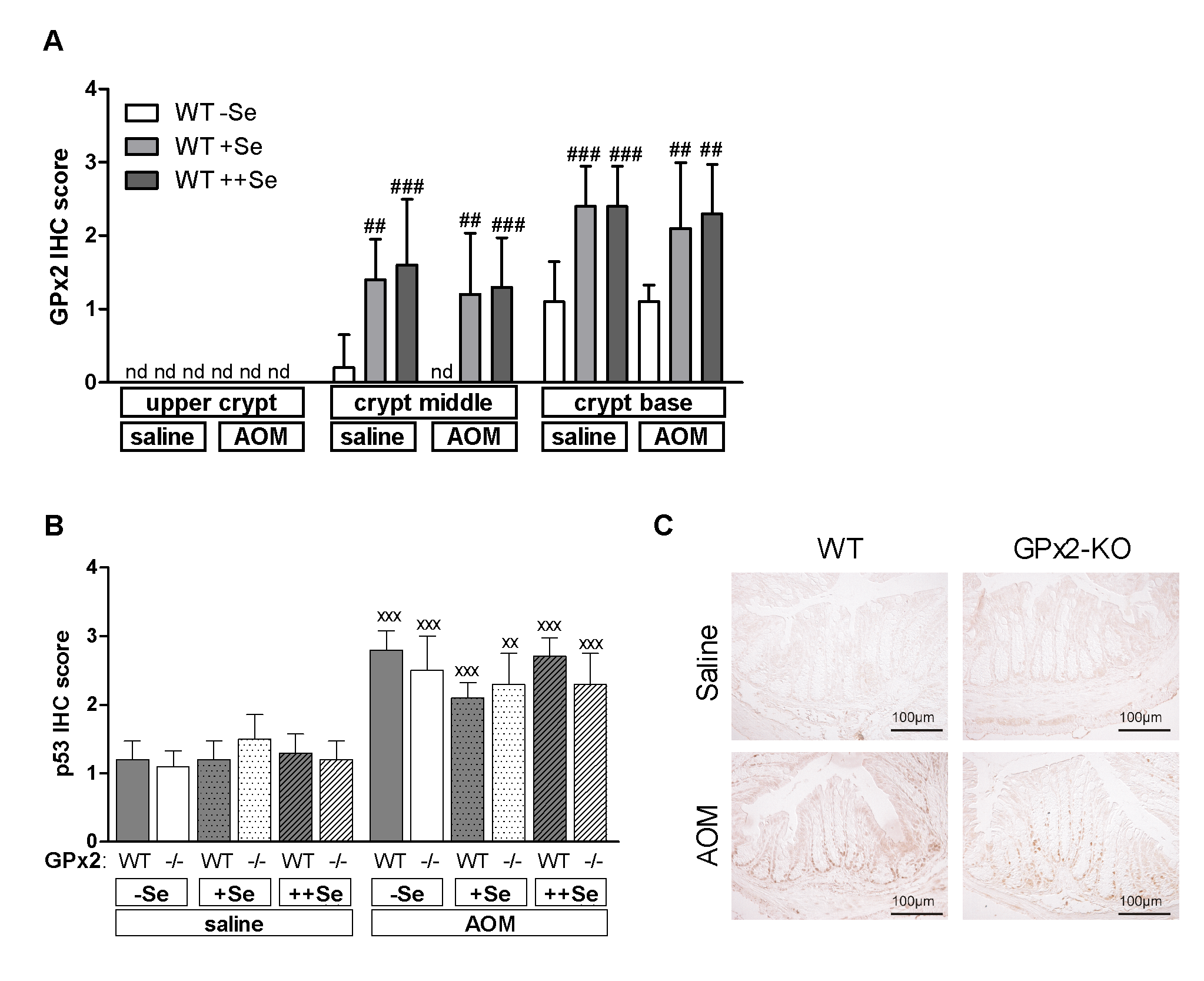

Supplement: Figure S1 — GPx2 and p53 response to AOM 8 h after single treatment. (A) GPx2 IHC staining was scored in the colon of WT mice fed the different selenium diets (nd = not detectable). (B) Nuclear staining of p53 was scored in WT and GPx2-KO mice. (C) Representative picture of p53 in -Se WT and GPx2-KO colon. Values are means +SD, n = 5. Significance was calculated using 2-way ANOVA with Bonferroni’s post-test. #P≤0.05, ##P≤0.01, ###P≤0.001 vs. -Se, xxP≤0.01, xxxP≤0.001 vs. saline. (TIF) [file pone.0072055.s001.tif]
